# TIGER: tiled iterative genome assembler

**DOI:** 10.1186/1471-2105-13-S19-S18

**Published:** 2012-12-19

**Authors:** Xiao-Long Wu, Yun Heo, Izzat El Hajj, Wen-Mei Hwu, Deming Chen, Jian Ma

**Affiliations:** 1 Department of Electrical and Computer Engineering, University of Illinois at Urbana-Champaign, Urbana, IL 61801, USA; 2 Department of Bioengineering, University of Illinois at Urbana-Champaign, Urbana, IL 61801, USA; 3 Institute for Genomic Biology, University of Illinois at Urbana-Champaign, Urbana, IL 61801, USA

## Abstract

**Background:**

With the cost reduction of the next-generation sequencing (NGS) technologies, genomics has provided us with an unprecedented opportunity to understand fundamental questions in biology and elucidate human diseases. *De novo *genome assembly is one of the most important steps to reconstruct the sequenced genome. However, most *de novo *assemblers require enormous amount of computational resource, which is not accessible for most research groups and medical personnel.

**Results:**

We have developed a novel *de novo *assembly framework, called Tiger, which adapts to available computing resources by iteratively decomposing the assembly problem into sub-problems. Our method is also flexible to embed different assemblers for various types of target genomes. Using the sequence data from a human chromosome, our results show that Tiger can achieve much better NG50s, better genome coverage, and slightly higher errors, as compared to Velvet and SOAPdenovo, using modest amount of memory that are available in commodity computers today.

**Conclusions:**

Most state-of-the-art assemblers that can achieve relatively high assembly quality need excessive amount of computing resource (in particular, memory) that is not available to most researchers to achieve high quality results. Tiger provides the only known viable path to utilize NGS *de novo *assemblers that require more memory than that is present in available computers. Evaluation results demonstrate the feasibility of getting better quality results with low memory footprint and the scalability of using distributed commodity computers.

## Background

Among scientific disciplines, genomics has one of the fastest growing bodies of data today. This is largely due to the recent advances in next-generation sequencing (NGS) technologies, which have tremendously reduced DNA sequencing costs. This massive amount of sequencing data have provided the basis to better understand the tree of life and to identify molecular signatures of human variation and disease mechanisms. To make such analyses possible, the key computational task is to *de novo *assemble raw reads from NGS technologies into complete or near-complete genomes. However, the enormous amount of data creates an inevitable barrier to the assembly process in terms of memory usage. In addition, the lower quality and limited read length produced by NGS, as compared to the traditional Sanger sequencing, make it extremely difficult to assemble reads into long scaffolds, which are essential to facilitate the analyses of large-scale genome rearrangements.

Most of the modern NGS-based *de novo *genome assemblers adopt the de Bruijn Graph (DBG) data structure to handle extremely high coverage data [1-3]. Several assemblers have specifically been developed with some success to assemble large genomes. In SOAPdenovo [4] and ALLPATHS-LG [5], a DBG was constructed in a large shared memory and the assembly process was done in parallel within multiple threads. However, all of them required hundreds of gigabytes (GB) of memory to assemble large genomes, such as those from human and other mammalian species. To tackle this problem, ABySS [6], YAGA [7] and PASHA [8] developed distributed DBG algorithms that split the DBG and parallelize the assembly process on a cluster on the basis of message passing interface (MPI). However, this imposed considerable communication among servers because many adjacent vertices in the DBG could be located on different servers. The amount of communication among servers increases nonlinearly when the number of servers increases, causing scalability issues. Some assemblers made modifications to DBG in order to reduce memory usage. Gossamer [9,10] used a compressed bitmap representation of the DBG, resulting in a memory usage that could be close to the theoretical minimum value of a DBG. Cortex [11] utilized colored DBG to detect the variations among 10 human genomes with less than 256 GB of memory. SparseAssembler2 [12] reduced memory usage dramatically by storing only a small fraction of *k*-mers. SGA [13] used a compressed index of reads and it could assemble a human genome under 60 GB of memory. Despite these developments, the memory usage of these tools is still too large for current commodity multi-core systems, limiting the scope of *de novo *assembly for large genomes to research groups that own large computer clusters. Therefore we urgently need new computational framework for scalable *de novo *genome assembly.

In this study, we made a key observation that the root of the NGS genome assembly problem in terms of memory usage and scalability could be solved if the large computational tasks could be decomposed into modest-sized independent sub-problems, which could then fit into smaller memories and be solved in parallel. This can effectively move large-scale *de novo *assembly tasks into commodity PC networks. In addition, when it is done right, this new approach would even lead to better assembly quality compared to the current state-of-the-art assemblers, as shown in detail later in this paper. We develop a highly effective framework for decomposing the problem of genome assembly from NGS reads. The decomposed sub-problems can be either solved in a sequential manner using significantly less memory or solved simultaneously if more computing nodes are available.

Besides the limitation on computing resources, several works have compared NGS *de novo *assemblers [14-20] and it is acknowledged that no assembler is the best across all applications and datasets. To deal with this issue effectively, our framework is designed in such a way that it can seamlessly embed different assemblers into the framework to take advantages of unique strengths of each assembler. None of existing assemblers can do this. These embedded assemblers work on decomposed sub-problems mentioned above efficiently. Through an iterative improvement approach facilitated by this framework, we are able to achieve higher assembly quality than the original assemblers themselves.

## Methods

Most assemblers can deal with small genomes (such as *E. coli*) very well using a small amount of computation resource and time. For very large genomes (such as mammalian-size genomes), most assemblers either cannot produce good results or require tremendous amount of resources and/or time. Besides, assemblers usually have their own design characteristics targeting at specific types of genomes [21]. Our approach aims to substantially reduce the computational complexity and resources needed for large genome assembly. The key innovation is to more effectively divide the genome assembly problem into smaller sub-problems and assemble them individually (without inter-node communication) with the flexibility of using various off-the-shelf assemblers. We use an iterative refinement approach to gradually improve the quality of problem partitioning and the overall solution.

### Key ideas

#### Tiled genome assembly

The rationale of our method follows our belief that the genome assembly could be done part-by-part instead of as a whole, namely the input reads can be divided into multiple tiles (or clusters) and the assembly results of all tiles can be merged as the final assembly. We call this approach *tiled genome assembly*. We observed that if we can have all related information (e.g., reads) for only a short fragment of a target genome, most assemblers would get excellent results and require much less memory. Including more reads that correspond to larger regions increases memory requirement and potentially makes assembly results worse. The main reason is that *de novo *assemblers cannot tell which part of the genome the reads belong to. However, if we can partition the reads in an effective way, assemblers can produce better results while requiring much less memory.

Taking the DBG-based assemblers as an example, ideally a contig (from a specific region in the target genome) is supposed to be built using only the *k*-mers extracted by the reads contributing to that region. However, most assemblers extract the *k*-mers from all the input reads and mix them together when constructing the DBG. More specifically, the *k*-mers whose source reads are not contributing to a specific region in the target genome may still be used in the DBG construction process. For such *k*-mers, we call them *ambiguous k-mers*. For genomes that are less repetitive, the ambiguous *k*-mers could be few. But for genomes that are highly repetitive, they can be significant enough to confuse the assembly process.

Therefore, we designed a new approach to partition the input reads into multiple tiles. Our goal is to have each tile contain only those reads contributing to a specific region of the target genome. The reads in such read tiles are called *well-clustered reads*. Thus the effect from ambiguous *k*-mers can be dramatically reduced. Since each read tile has all the necessary information, no communication would be needed among the assemblies of different read tiles. Since regions in the target genome can be assembled independently and as a result, each assembly will need less memory to complete.

#### Read clustering based on clustered contigs

A well-clustered read tile should contribute to a continuous region of the target genome. The region can be composed of one long contig or a set of contigs covering the whole region without gaps in-between. A set of such contigs is called *well-clustered *and can be obtained by sorting or *clustering *closely related contigs together. Therefore, by aligning the input reads against a well-clustered contig set, the reads having high similarity with sub-sections in the contigs can be collected as a well-clustered read tile. This process is called *read clustering*. The collected reads can then be assembled to produce a similar set of the contigs but with improved contig lengths and quality.

#### Intermediate reference genome

A target genome can be considered as a combination of multiple continuous regions (the minimum number is the number of chromosomes in the target genome), where each region can be contributed completely by one or many contigs. Therefore, ultimately there would be multiple well-clustered contig sets corresponding to multiple regions in the target genome. In our approach, we treat the contigs from assembly as the *intermediate reference genome *and arrange the contigs in multiple clustered contig sets.

For *de novo *assembly, we start from random partitions of the reads in tiles, assemble the reads in each tile, and merge all contig sets into one as the intermediate reference. In this case, the reads in an initial, randomly partitioned tile will correspond to random regions in the target genome. As a result, the initial contig sets that serve as the intermediate reference will likely be fragmented and will have errors. Our approach iteratively improves the clustering and thus the quality of the intermediate reference genome. In the end, the intermediate reference genome converges to the final target genome.

#### Iterative assembly

The transformation from reads to contigs and from contigs to reads forms a cycle. Thus the whole assembly flow can be *iterative*. As more transformation iterations are performed, contigs become longer with higher quality since read clustering improves, and each tile contains less irrelevant information that may confuse the assembly process.

### The Tiger algorithm

**Figure 1:**
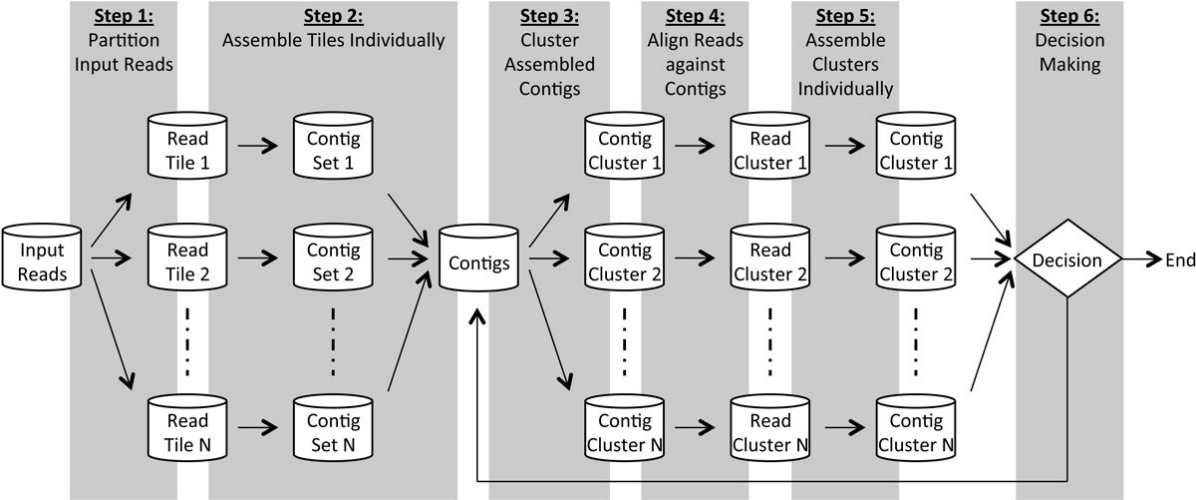
**Schematic view of our iterative framework for genome assembly**.

Based on aforementioned ideas, we developed "Tiled Iterative GEnome assembleR," or Tiger. Here "tile" is a synonym of "set" or "cluster", representing the tiled computation nature in the assembly process. The conceptual flow is illustrated in Figure 1.

**Step 1. Reads partitioning: **We first partition the input reads into multiple read tiles. In our current implementation, the input reads are randomly partitioned evenly into *N *subsets, which can be determined by users based on the available resources and the total size of the input reads.

**Step 2. Read assembly: **Read tiles are assembled individually, using an off-the-shelf assembler, such as Velvet, embedded into Tiger. Depending on the available system memory, the assembly of read tiles can be done independently in serial or in parallel on a shared or distributed memory computer cluster. There is no communication between the assemblies of different read tiles.

For the embedded assembler requiring specifying a *k*-mer size, *k*-mer sizes are decided either manually by users or automatically through the auto-*k*-mer scheme in Tiger. For the manual *k*-mer designation, a *k*-mer size is used in all read tile assemblies for all Tiger iterations. Otherwise, the auto-*k*-mer scheme randomly picks *k*-mer sizes within a given range and records the best *k*-mer size in the assembly results. The best *k*-mer size and the randomly picked ones will be considered in the subsequent assemblies. User-specified *k*-mer size can be introduced into this *k*-mer history database but may not be used again if the first attempt is not good. The number of used reads in the assembly, the total length of the contigs, and the resultant N50s are used to evaluate whether a *k*-mer size can help produce the best result without knowing the target genome. This avoids the problem of picking a contig set with high N50 and low coverage and enables Tiger to find a good direction in the iterative process and to converge to high quality results.

Since Step 2 is the first time to assemble the initial read tiles, the contigs can be short and may cause long running time in the later iterations. We address this issue by merging the contig sets and feed the merged contig set to Velvet with the LONGSEQUENCE flag enabled. Velvet may further elongate the contigs by treating the input contigs as long reads. The new contig set is used when it is better than the merged contig set. The output contig set is scaffolded by SSPACE [22]. The scaffolded contig set is the input to Step 3. The purpose of this scaffolding process is to leverage paired-end information to bridge contigs which may be from different assemblies. This is beneficial for better clustered contigs at Step 3. The scaffolding process also helps resolve duplicated contigs from different assemblies.

**Figure 2:**
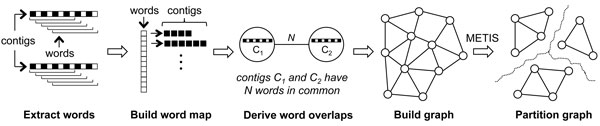
**Contig clustering algorithm**. Words are extracted from contigs. The number of common words between two contigs is used as the edge weight in the graph. Contig lengths are modeled as vertex weights. The contig connectivity graph is thus built, followed by the METIS partitioning process. The partitioned sub-graphs are clustered contig sets.

**Step 3. Contig clustering: **The overall contig clustering algorithm is depicted in Figure 2. A graph that models the contig connectivity intensity is built from the merged contig set. This graph is called the *contig connectivity graph*. Graph vertices are the contigs. Vertex weights are contig lengths. Edge weights are defined based on the contig overlapping degree with each other. Note that the contig connectivity graph is much smaller than the DBG so it uses much smaller amount of memory. We then apply a graph-partitioning tool, METIS [23], to partition the graph into contig clusters. METIS is known to be fast and memory-efficient in processing millions of graph vertexes.

The contig lengths (vertex weights) are given less importance than the contig overlapping degrees (edge weights) in the graph partitioning process. This is because we want the partitioned contig connectivity sub-graphs to be more edge-oriented instead of being vertex-oriented. But we still need to consider the vertex weights for the situations where there exist many short contigs with little connectivity in-between. This is very common for the assembly results in the first few iterations on assembling randomly partitioned read tiles. These short contigs ought to be distributed to all clusters evenly. This not only preserves their existence in the following Tiger iterations but also reduces their influence on the rest of the clustered contigs.

By focusing graph partitioning on edge intensity, overlapping contigs will be grouped together and would be rebuilt as one long complete contig later at Step 5. These contigs are used to produce well-clustered reads in the read clustering process at Step 4. That is, this contig clustering step makes crucial contribution to the quality of results of the later steps.

Building of a contig connectivity graph can be time-consuming if a traditional sequence alignment method is used, like the Smith-Waterman algorithm [24]. Since the degree of overlap between contigs need not be determined exactly for our purposes, we apply a heuristic algorithm based on the image recognition algorithm using vocabulary trees in [25], with inverse document frequency scoring dropped. We begin by extracting consecutive sequences of equal length (called *words*) from each of the contigs in the set. The extracted words are used to build a map (or inverted file) from the words to the contigs containing them. A contig connectivity graph is then built from the map with the edge weights being set to the number of words in common between the vertices (contigs) connected by that edge. Since the connectivity graph stores only the contig connectivity information, the memory usage of this step is much lower than that at the read assembly step. Regarding runtime, building a connectivity graph dominates the whole step. Building the word map can be done in parallel but we leave it as future work. Overall the runtime is still much shorter than Step 4 and 5.

**Step 4. Read clustering: **The entire input read set is aligned to the contig sets from Step 3. The reads having high similarity to each contig set are collected as one read cluster. Each read is collected once for each cluster. This means a read can appear in multiple read clusters if similar contigs are not clustered together. This process guarantees any read potentially contributing to a contig set will be collected. The read-to-contig alignment is done by Bowtie [26]. For paired-end reads, if one of a read pair aligns to the given contig cluster, both reads are collected. This step provides the opportunity to extend and/or bridge the contigs. This clustering process can be done in parallel or in serial on a shared or distributed memory computer cluster. No communication is needed between read tiles. The required memory is also proportional to the size of a read tile.

**Step 5. Read assembly: **We assemble the read tiles, the same as we do at Step 2. But the assembly of the merged contigs from all read tile assemblies may not be performed. If the assembly of the merged contigs is not improving, it is skipped in later iterations to save time. Based on our experience, we found it is useful to have this additional assembly in the first few iterations.

**Step 6. Post processing: **If we have reached the given number of iterations, we will just exit. Otherwise, go to Step 3. Step 3, 4, and 5 form an iterative process.

To sum up, the rationale behind our framework is that the improvement on contig quality of the current iteration can be carried over to the next iteration through more accurate read clustering. An optimal clustering solution will be achieved if only reads contributing to a contig are clustered for assembling the contig. This approach differentiates our algorithm from the previous work and provides our framework the capability of improving an existing contig set further.

## Results

### Evaluation environment setup

Two well-known assemblers were embedded into Tiger for this evaluation, i.e. Velvet [3] (version 1.2.03, compiled with max *k*-mer 96 and 4 categories) and SOAPdenovo [4] (version 1.05), named as Tiger-Velvet and Tiger-Soap, respectively.

Two types of evaluation were carried out: type R and type I, labeled as Tiger-Velvet/Soap-R/I. Type R (Random) started from the randomly partitioned multiple read tiles followed by Tiger-Velvet/Soap, which demonstrated that Tiger can manage the randomly partitioned read tiles and gradually improve the result to achieve better NG50 than the corresponding single-tile assembly (i.e., the solution provided by the original assembler itself). Type I (Improved) started from the assembly result generated by Velvet/SOAPdenovo (instead of random partitioning), followed by Tiger-Velvet/Soap, respectively, to improve the result. This was to demonstrate that Tiger could also improve the single-tile assembly by its embedded assembler. Both types of evaluation used 150-tile assembly with auto-*k*-mers.

The machine for these evaluations is installed with Intel Core i7 CPU 950 (4 physical cores in clock rate 3.07 GHz), 24 GB system memory, and 2 TB disk space. Five of such machines were used.

### Data used

The human chromosome 14 data set in the GAGE assembly competition [20] was mainly used to assess Tiger. The chromosome length is 88 Mbp excluding Ns. The data set details are summarized in Table 1. Same as [20], the reads were corrected by Quake [27] before assembly. The other data set was the 4.6 Mbp long *E. coli *genome (Illumina paired-end reads, accession no. SRR001665) with 36 bp read length, generated from a 200 bp insert length, *E. coli *K-12 MG1655 library (accession no. NC_000913). The assembly results were analyzed by the evaluation script from [28], using the MUMMer package [29], with 200 bp as the minimum contig length.

**Table 1:** Details of the human chromosome 14 read libraries.

Genome size (bp)	Read library 1		Read library 2		Read library 3	
	
	# of reads	Insert length	# of reads	Insert length	# of reads	Insert length
88,289,540	32,621,862	155	14,054,994	2,280-2,800	2,009,674	35,000

The same analysis metrics in [20] are reused in Table 2 and Table 3. The NG50 value is the smallest contig size such that 50% of the reference genome is contained in contigs of size NG50 or larger. The error-corrected NG50 is calculated by splitting contigs at every misjoin and at every indel that is longer than 5 bp. SNPs mean the single nucleotide differences. Inversions are the reversed sequences in strands. Relocations are the sequence rearrangements. "Unaligned ref." is the bases in the reference that was not aligned to any contig. "100% - Unaligned ref." is the genome coverage. "Duplicated ref." is the sequence occurrence frequencies in contigs.

**Table 2:** The human chromosome 14 assembly results in terms of continuity, accuracy, and statistics.

Evaluations	Continuity			Accuracy			Statistics		
	
	Contig #	NG50 (kbp)	NG50 corr. (kbp)	SNP	Indels	Misjoins	Asm. (%)	Unaligned ref. (%)	Duplicated ref. (%)
Velvet 61k	28,974	5.2	4.7	82,235	17,755	601	96.69	2.09	0.43

Tiger-Velvet-R 125i	20,189	11.6	9.3	84,577	21,847	533	97.90	1.98	1.50

Tiger-Velvet-I 7i	21,623	10.9	8.9	84,811	21,470	654	98.43	1.53	1.48

SOAPdenovo 55k	50,094	3.0	3.0	67,956	11,866	36	95.91	3.13	0.28

Tiger-Soap-R 120i	60,134	3.6	3.4	68,881	12,839	185	99.40	3.01	2.79

Tiger-Soap-I 7i	55,173	3.8	3.6	69,215	13,390	205	98.68	2.43	1.46

The columns include the number of contigs, NG50 size and its error-corrected size, the number of single nucleotide polymorphisms (SNPs), the number of indels and misjoins in contigs, total assembly length, genome coverage (100 - Unaligned ref.), and duplications. K-mer 61 and 55 are the best *k*-mer sizes for Velvet and SOAPdenovo, respectively. "#k" stands for the applied k-mer size. "#i" stands for the iteration number.

**Table 3:** *E. coli *(SRR001665) 24-tile assembly results in terms of contiguity, accuracy, and statistics.

Evaluations	Continuity			Accuracy			Statistics		
	
	Contig #	NG50 (kbp)	NG50 corr. (kbp)	SNP	Indels	Misjoins	Asm. (%)	Unaligned ref. (%)	Duplicated ref. (%)
Velvet 25k	147	87.0	67.3	238	37	2	97.89	0.56	0.01

Tiger-Velvet-R 51i	281	95.6	87.2	190	35	3	100.92	0.14	2.30

Tiger-Velvet-I 7i	276	95.4	87.2	211	33	12	100.40	0.12	1.68

SOAPdenovo 27k	450	17.9	17.9	12	4	1	97.56	1.31	0.00

Tiger-Soap-R 80i	524	25.6	25.6	31	11	3	98.67	1.20	0.78

Tiger-Soap-I 7i	509	25.8	25.8	23	6	2	98.78	0.80	0.64

The columns include the number of contigs, NG50 size and its error-corrected size, the number of single nucleotide polymorphisms (SNPs), the number of indels and misjoins in contigs, total assembly length, genome coverage (100 - Unaligned ref.), and duplications. K-mer 25 and 27 are the best *k*-mer sizes for Velvet and SOAPdenovo, respectively. "#k" stands for the applied k-mer size. "#i" stands for the iteration number. Both Tiger-Velvet-I and Tiger-Soap-I evaluations use the best results from Velvet and SOAPdenovo as input, respectively.

### Evaluation results

Table 2 and Table 3 summarize the evaluation results. More detailed results are listed in Appendix: Tables. For human chr14 data, we show that Tiger results have better NG50s and genome coverage as compared to the best Velvet and SOAPdenovo results using *k*-mer sizes 61 and 55, respectively, indicating that Tiger can iteratively improve the assembly results. To demonstrate that Tiger can improve not only the best single-tile assembly result but also a common one by its embedded assemblers, Tiger-Velvet-I used the Velvet best result as input and Tiger-Soap-I used the SOAPdenovo result with *k*-mer size 61. It is noted that although Tiger applies an iterative assembly process, with the same parameter setting and inputs, Tiger can reproduce the same results.

The NG50s and coverages of the type R show continuous improvement from iteration to iteration. The best NG50s by Tiger-Velvet/Soap-R reach 11.6 kbp and 3.6 kbp (or 2.2x and 1.2x improvement), respectively, as compared to the best Velvet/SOAPdenovo results. The type I results also show continuous NG50 improvement. Regarding the coverages, although Tiger-Velvet-I results have an improving trend, such trend is not clear on Tiger-Soap-I results. The best NG50s by Tiger-Velvet/Soap-I reach 10.9 kbp and 3.8 kbp (or 2.1x and 1.3x improvement), respectively.

As for the accuracy, the best Tiger-Velvet/Soap results of both R and I flows had higher SNPs and indels errors. The misjoin errors by the best Tiger-Velvet-R result were less. But the best results of Tiger-Velvet-I and Tiger-Soap-I/R had higher misjoin errors. We suspect this is because the read clustering step has collected some irrelevant reads due to unresolved duplications. Note that, in the *E. coli *results, both Tiger-Velvet/Soap produced similar misjoin errors against their counterparts. This suggests that the higher error rate in Tiger is also related to the reads characteristics.

Table 4 lists the runtime and memory usage results on the read assembly to demonstrate the low memory usage of tiled assembly using multiple auto-*k*-mers. Tiger-Velvet consumes the least amount of memory as low as 0.16 GB. On the other hand, Tiger-Soap still consumes 1.8 GB even though the read tile file size is around 10 MB only, whereas the 1-tile read file size is 4.7 GB. The runtime between the evaluations by Velvet/SOAPdenovo and Tiger-Velvet/Soap shows that Velvet and SOAPdenovo run much faster when the read tile size is small. For instance, the runtime for the 150-tile Tiger-Velvet-R assembly with 8 auto-*k*-mers is less than twice of the 1-tile Velvet assembly. The runtime between the Tiger-Velvet/Soap evaluations with 3 and 8 auto-*k*-mers shows that embedded Velvet and SOAPdenovo take more time and memory for better-clustered read tiles because the contigs in a DBG can be assembled further by the clustered reads. However, for less-clustered read tiles, the contigs are shorter in a DBG with smaller memory and the assembly ends earlier.

**Table 4:** The runtime and memory usage of the assemblies on the human chromosome 14 genome.

Evaluations	Wall-clock Time (Hr.)	Peak memory usage (GB)	Thread # in total	K-mer size #	Tile #
Velvet 61k	0.95	8.26	1	1	1

Tiger-Velvet-R 1i	1.49	0.16	1	8	150

Tiger-Velvet-I 1i	1.96	0.29	1	3	150

SOAPdenovo 55k	0.43	8.31	1	1	1

Tiger-Soap-R 1i	1.35	1.8	1	8	150

Tiger-Soap-I 1i	1.67	1.9	1	3	150

All evaluations are done using 1 thread. "#k" stands for the applied *k*-mer size. "#i" stands for the iteration number. Note: The runtime and memory usage for Tiger is on the read assembly (Step 5) only.

Table 5 further lists the detailed computational resource usage using different numbers of threads across computers by Tiger and its counterparts. The runtime and memory usage include the whole Tiger assembly process from Step 3 to 5. Since the resource usage of a Tiger iteration can be very different especially for the type R tests, we used the first iteration of the type I because it is stabilized and consumes more resources than the type R iterations. The peak memory usage by Tiger using one thread was 1.87 GB and the runtime went to 4.69 hours. The 1.87 GB memory is from the contig clustering (Step 3) because the current implementation targets at 4 GB memory machine. The memory usage of 4-thread execution was 2.44 GB. This demonstrates Tiger's capability of running on commodity computers.

**Table 5:** Comparison of the runtime and memory usage on the human chromosome 14 assembly.

Evaluations	Wall-clock time (Hr.)	Speedup against 1 thread	Peak memory usage (GB)	Thread # in total	Machine #	K-mer size #	Tile #
Velvet 61k	0.95	1x	8.26	1	1	1	1

Velvet 61k	0.47	2.02x	8.40	4	1	1	1

SOAPdenovo 55k	0.43	1x	8.31	1	1	1	1

SOAPdenovo 55k	0.25	1.72x	8.50	4	1	1	1

Tiger-Velvet-I 1i	4.69	1x	1.87	1	1	1	150

Tiger-Velvet-I 1i	1.58	2.98x	2.44	4	1	1	150

Tiger-Velvet-I 1i	0.83	5.69x	N/A+	12	3	1	150

Tiger-Velvet-I 1i	0.66	7.16x	N/A+	20	5	1	150

"#k" stands for the applied *k*-mer size. "#i" stands for the iteration number.

+ The memory usage across machines can not be measured in our environment.

**Figure 3:**
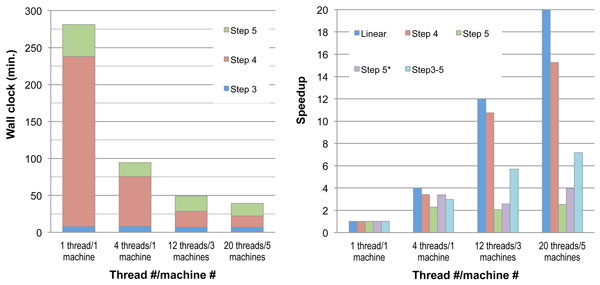
**Tiger-Velvet-I 1i runtime comparison using the human chromosome 14 data**. Different numbers of threads across machines are used. The speedup base line is labeled as 1x for other corresponding columns. The *k*-mer size 61 is used in all tests to avoid varying runtime caused by different *k*-mer sizes. Step 5* does not include SSPACE result since it does not execute across computers.

When more threads across computers were given, the runtime speedup were 2.98x, 5.69x, and 7.16x, which are not proportional to the linear speedup, 4x, 12x, and 20x, respectively with the given thread numbers (4, 12, and 20). Since there were unparallelized parts, we dissected Tiger into steps with individual timing information, as shown in Figure 3. For the 1-thread evaluation, Step 4 took up to 81.86% out of all three steps 3, 4, and 5 since our current implementation is not optimized yet. Step 5 took 15.30%. However, Step 4 performs read-to-contig alignments, where the runtime of alignment tasks is similar to one another. This fits best the bulk-synchronous-parallel computation model so the speedup numbers were 3.41x, 10.74x, and 15.26x, showing close to linear results of the speedup, 4x, 12x and 20x, respectively. At Step 5, the bulk-synchronous-parallel computation model is also used. The last contig scaffolding task was parallelizable within one computer so when the scaffolding task was in progress, the other computers were idle. However, although the rest of the tasks were mostly parallelizable, the runtime speedup was still not linear. This is because the assembly time of each read tile is very different from one another such that unbalanced load takes place often, meaning many threads were idle, waiting for the last one to finish. This suggests the bulk-synchronous-parallel model may not work well for Step 5 on parallel read assemblies. Overall, there is still much room for future work to further accelerate our framework.

For the Velvet and SOAPdenovo evaluations, the memory usage did not change much when more threads were added. When 4 threads were used, the runtime speedup for Velvet and SOAPdenovo were 2.02x and 1.72x, respectively. No tests on multiple computers were carried out since neither assemblers could execute across computers with distributed memory. Although the assemblies using the best *k*-mers consumed about 8.5 GB, locating the best *k*-mers required enumerating all possible *k*-mers which actually required more than the 24 GB memory on the machine we did our tests, e.g. *k*-mer 37 for Velvet. We used a computer cluster with 2 TB memory to overcome the memory explosion in assemblies. On the other hand, Tiger did not have this problem since in our evaluations each read tile size was about 1/150 of the input reads. This shows the advantage of Tiger when a 2 TB memory machine is not attainable.

## Discussion

### Choice and effect of the number of read tiles

The choice of the number of read tiles affects not only the processing time for good results but also the quality of results the assembler can reach. The more the number of read tiles, the longer processing time it will take and the better quality result the assembler can reach. Since in the beginning iterations contigs are shorter and have less overlap with one another, the transformation between reads and contigs needs more time to converge. When read tiles reach a well-clustered state, the assembler can focus on a smaller set of the read information and produce better quality results.

### Choice of *k*-mers for read tiles

The choice of *k*-mer sizes for DBG-based assemblers is an unresolved issue. This is important because it has noticeable impact on the assembly results. Related works [2,30,31] either explicitly iterate all possible *k*-mer sizes or implicitly find suitable ones at different levels of assembly granularities for best results. In Tiger, the input reads are arranged in multiple relatively small clustered read tiles such that these *k*-mer size searching algorithms can provide better results. For the assemblers that require specifying *k*-mer sizes, the auto-*k*-mer scheme in Tiger picks the best *k*-mer sizes for each read tile. Based on our experience, the best *k*-mer sizes selected by the auto-*k*-mer scheme for each read tile are actually not the best ones used by the best single-tile assemblies. For example, in the Tiger-Velvet-R, the selected top three *k*-mer sizes were actually 55, 57, and 59 instead of the best *k*-mer size 61 by Velvet.

### Novel features in Tiger

**Iterative improvement of assembly results**: To the best of our knowledge, none of existing methods can provide this functionality. The iterative transformation between contigs and reads gradually improves the read clustering quality and thus assemblers need to manage only a smaller portion of the original read information. In the iterative process, the scale and complexity of the assembly problems are reduced.

**Low memory footprint of each read tile assembly**: Our focus is on decomposing the input reads into sub-assembly problems, instead of decomposing the de Bruijn graph data structure. The required memory for an assembly is inversely proportional to the number of read tiles. When the read coverage exceeds the number of read tiles, the assembly of each tile may need large amount of memory in the beginning iterations due to random reads partitioning. This is because each read tile may contain considerable proportion of all *k*-mers for the whole target genome assembly. We can increase the read tile number to make the memory of each read tile assembly acceptable.

**Assembler embedding**: It is known that every assembler has its characteristics for specific types of genomes [21]. The input to our framework can be an assembly result from assembler A. Tiger can embed assembler A to further improve it, as what is demonstrated in our evaluations. In addition, Tiger can also embed another assembler B to improve the results done by A.

**Scalable parallel assembly process**: In Tiger, the most time consuming steps are read clustering and read assembly. Both are highly parallel and do not need communication between threads. This makes the framework suitable for either distributed or shared memory computer clusters.

### Drawbacks in Tiger

**More duplications**: In the results, we saw more duplications in Tiger assemblies. For a single-tile assembly, assemblers usually can detect duplications and resolve some of them. Since in Tiger, assemblies for tiles are done independently, duplications are more likely to take place. The is because when the duplications are in the contig ends, scaffolding tools usually can resolve them by merging contigs together. For example, the scaffolding tool SSPACE can help eliminate contig-end duplications. When the duplications are in the middle of two contigs, we believe a post-processing step can resolve them, which will be our future work.

**Shorter scaffolds**: We did not show the scaffold results because currently at the contig clustering step, contigs are clustered only based on the degree of overlap with one another, but the contigs that can be scaffolded are not taken into consideration. We will add a scaffolding phase to Tiger in our future development.

## Conclusions

We developed a novel methodology for sequence assembly, providing comparable or better quality results while adapting to available memory resources. The key idea behind our approach is partitioning and clustering reads so that the memory usage for assembly is inversely proportional to the number of partitions. This fundamentally resolves the issue of high memory requirement in sequence assembly and potentially accelerates the research progress of this field. Our approach can leverage existing well-built assemblers and iteratively improve the results. Our approach can also start from an already assembled result and further improve it, using the original read set. To the best of our knowledge, none of works so far has this capability. In situations where traditional assemblers require more memory than that is present in available computers, our approach provides the only known viable path. Evaluation results demonstrate the feasibility of getting better quality results with low memory footprint and the scalability of using distributed commodity computers. The Tiger program can be downloaded from http://impact.crhc.illinois.edu/.

## Competing interests

A provisional patent application that includes portions of the research described in this paper has been filed by the Office of Technology Management at the University of Illinois at Urbana-Champaign.

## Authors' contribution

XLW initiated the idea. WMH, DC, and JM conceived the research. XLW developed most of the source code and carried out the evaluation of the tool. YH and IEH participated in the code development and data analysis. XLW drafted the manuscript with help from YH and IEH. WMH, DC, and JM revised the manuscript. All authors read and approved the final manuscript.
